# Medical Classes

**Published:** 1879-10

**Authors:** 


MEDICAL CLASSES.—Just as this number of the JOURNAL AND EXAMINER goes to press, the regular medical colleges in this city are commencing the active work of another collegiate year. We learn from reliable sources that the number of matriculants already registered in each, indicates a full attendance and a prosperous term. This is as it should be, for we are sure that the colleges here are as well prepared to give the student as thorough and complete instruction in all the departments of medicine and surgery; and the faculties are as faithful in exacting an honest fulfillment of their requirements, as those of any colleges in this country.

---

A URINE THERMOMETER FOR GYNÆCOLOGICAL PRACTICE.—Dr. Otto Küstner, of Jena, describes an ingeniously arranged instrument composed of a silver female catheter which contains in its lumen a self-registering thermometer. The catheter tapers sharply below the point to which the bulb of the thermometer reaches, and the instrument is so arranged that the thermometer can be removed if desired. The urine in passing out bathes the thermometer, and only fifteen seconds are required to bring the temperature up to the maximum, which is the same as that in the axilla, or within a fraction of it (0.3°–0.5° C. higher).—*N. Y. Medical Record.*

